# Treatment of Cardiac Fibrosis with Extracellular Vesicles: What Is Missing for Clinical Translation?

**DOI:** 10.3390/ijms241310480

**Published:** 2023-06-22

**Authors:** Sebastian Neuber, Miriam R. Ermer, Maximilian Y. Emmert, Timo Z. Nazari-Shafti

**Affiliations:** 1 Department of Cardiothoracic and Vascular Surgery, Deutsches Herzzentrum der Charité (DHZC), 13353 Berlin, Germany; miriam.ermer@dhzc-charite.de (M.R.E.); maximilian.emmert@dhzc-charite.de (M.Y.E.); timo.nazari-shafti@dhzc-charite.de (T.Z.N.-S.); 2 Charité-Universitätsmedizin Berlin, Corporate Member of Freie Universität Berlin and Humboldt-Universität zu Berlin, 13353 Berlin, Germany; 3 BIH Center for Regenerative Therapies (BCRT), Berlin Institute of Health at Charité-Universitätsmedizin Berlin, 13353 Berlin, Germany; 4 German Centre for Cardiovascular Research (DZHK), Partner Site Berlin, 13353 Berlin, Germany; 5 Institute for Regenerative Medicine, University of Zurich, 8044 Zurich, Switzerland

**Keywords:** heart failure, cardiac fibrosis, extracellular vesicles, therapy, clinical translation

## Abstract

Heart failure is the leading cause of morbidity and mortality and currently affects more than 60 million people worldwide. A key feature in the pathogenesis of almost all forms of heart failure is cardiac fibrosis, which is characterized by excessive accumulation of extracellular matrix components in the heart. Although cardiac fibrosis is beneficial in the short term after acute myocardial injury to preserve the structural and functional integrity of the heart, persistent cardiac fibrosis contributes to pathological cardiac remodeling, leading to mechanical and electrical dysfunction of the heart. Despite its high prevalence, standard therapies specifically targeting cardiac fibrosis are not yet available. Cell-based approaches have been extensively studied as potential treatments for cardiac fibrosis, but several challenges have been identified during clinical translation. The observation that extracellular vesicles (EVs) derived from stem and progenitor cells exhibit some of the therapeutic effects of the parent cells has paved the way to overcome limitations associated with cell therapy. However, to make EV-based products a reality, standardized methods for EV production, isolation, characterization, and storage must be established, along with concrete evidence of their safety and efficacy in clinical trials. This article discusses EVs as novel therapeutics for cardiac fibrosis from a translational perspective.

## 1. Introduction

The cardiovascular field is in desperate need of translational success stories. Despite significant developments in pharmacological and device-based therapies to preserve cardiac output and delay disease progression in heart failure patients, there remains an enormous medical and financial burden, and innovative treatment strategies are urgently awaited [1]. Over the past twenty years, thousands of peer-reviewed articles and hundreds of pre-clinical and clinical trials have been published in the search for curative therapy for heart failure. However, they all have one thing in common: none of them have resulted in a clinical-grade product approved by a major regulatory authority. 

A central factor in the progression from acute myocardial infarction to chronic or terminal heart failure is cardiac fibrosis, which is characterized by extensive remodeling of the myocardial extracellular matrix (ECM) [2]. Although this mechanism is essential to maintain the structural and functional integrity of the damaged heart, unrestrained cardiac fibrosis can result in tissue stiffening and decreased ventricular filling and contraction, ultimately contributing to the development of heart failure, arrhythmia, and sudden cardiac death [3,4]. Conventional therapies, such as renin–angiotensin–aldosterone system inhibitors and β-blockers, have been shown to effectively reduce ECM protein deposition in the injured myocardium, but they do not completely prevent the progression of cardiac fibrosis in patients with heart failure [5]. Unfortunately, to the best of our knowledge, there are currently no approved therapies that specifically and effectively target cardiac fibrosis. Barriers to the development of treatments specific to cardiac fibrosis include (i) the molecular mechanisms underlying cardiac fibrosis, which are complex and not fully understood yet [6], (ii) the limited regenerative potential of the adult human heart after myocardial infarction, which does not allow complete inhibition of cardiac fibrosis; otherwise, there is a high risk of cardiac rupture [7], (iii) the volatile microenvironment in the injured heart, which is associated with increased levels of inflammation and cell proliferation, may compromise efficient delivery of therapeutics [8], and (iv) the scarcity of suitable in vitro and in vivo models that robustly recapitulate cardiac fibrosis in humans. 

In recent decades, cell-based approaches have been proposed as promising strategies to alleviate excessive cardiac fibrosis and improve heart function, but their clinical translation is complicated. Major challenges include the induction of innate or adaptive immune responses, the potential for tumor formation, and the low survival rate of transplanted cells at the targeted site [9,10]. Recognizing that the effect of administered stem/progenitor cells in myocardial injury is primarily mediated by the release of extracellular vesicles (EVs), EVs have attracted increasing attention due to their significant advantages in terms of stability, biocompatibility, and regulatory aspects [11]. The aim of this study is to outline the current prospects and challenges associated with cell-free EV-based products for the treatment of cardiac fibrosis, particularly from the perspective of clinical translation.

## 2. How Are EVs Defined and Where Do We Stand in the Regulatory Landscape?

The term EV, as coined by the International Society of Extracellular Vesicles (ISEV), includes all extracellular membrane-enclosed vesicles [12,13]. Structurally, EVs are nanoscale cell-derived particles with a lipid bilayer membrane that are secreted by most mammalian cells under both physiological and pathological conditions [14]. They can contain hundreds or thousands of bioactive molecules, including proteins, metabolites, lipids, and nucleic acids [15]. Currently, the EV field is one of the fastest-growing scientific areas, with more than 19,000 EV-focused articles found on PubMed, of which more than 80% have been published in the last 5 years. In addition, Clinicaltrials.gov lists more than 150 entries with “extracellular vesicles” as the search term. Due to their natural origin, EVs have several desirable properties, such as low immunogenicity and toxicity, high stability, excellent biocompatibility, inability to self-replicate, flexibility in dosing, and feasibility for pre- and post-isolation modification [16,17,18,19,20]. They can also overcome many of the limitations associated with current drug delivery systems, as they can cross biological barriers, travel long distances in body fluids, and deliver their cargo directly into the cytosol of recipient cells via membrane fusion and endocytosis [21]. In addition, EVs can be stored frozen for long periods of time to be available for immediate use in patients without significant loss of functional activity [22,23]. 

In order to successfully translate EV-based therapeutics to clinical practice, their quality, safety, and efficacy must be demonstrated, as is the case for any medicinal product. From a regulatory perspective, according to the guidelines of the European Medicines Agency (EMA) and the United States Food and Drug Administration (FDA), the classification of EVs depends on the specific therapeutic cargo they carry. In Europe, EVs are considered biologics if they are purified from non-modified cells or genetically engineered cells, but where the vesicles contain only functional transgenic protein. In contrast, when EVs are used as a delivery system for functional transgenic RNA with an intended therapeutic function in the patient, such a product is classified as an advanced therapy medicinal product and is subject to additional regulatory requirements [24]. The same criteria would apply in the United States [25]. Remarkably, while the EMA typically requires knowledge of a drug’s mechanism of action as part of the approval process [26], the FDA does not mandate such an understanding, only safety and some level of efficacy, meaning that entering direct clinical trials without this knowledge may be a potential path forward for EVs. However, a lack of understanding of EV-based products could lead to adverse outcomes, off-target effects, or ineffective dosing, which may explain why there is currently no EMA/FDA-approved clinical product containing eukaryotic EVs.

## 3. How Can EV-Based Products Interrupt Cardiac Fibrosis?

In general, fibrotic processes in the heart can be broadly divided into two categories: reactive and reparative fibrosis [27]. Reactive fibrosis describes the excessive accumulation of ECM components in the interstitial or perivascular spaces, triggered by pressure or volume overload or other pathological stimuli, and can result in impaired relaxation and filling of the heart ventricles after contraction [28]. Reparative fibrosis is classically associated with acute myocardial injury, in which damaged cardiomyocytes are replaced by ECM components to prevent cardiac rupture while maintaining the contractile function of the heart [29,30]. Consequently, EV-based products that induce complete suppression of cardiac fibrosis in patients with acute myocardial infarction may lead to serious side effects, such as ventricular aneurysms or fatal heart rupture. In these patients, the initial cardiac fibrotic response in the infarct area is necessary, but avoiding cardiac fibrosis in the infarct border zone and the surrounding or even distant myocardial tissue is a critical step in preventing subsequent heart failure [31]. 

At the cellular and molecular levels, there are a number of signaling pathways and mediators involved in cardiac fibrosis that may provide suitable therapeutic targets for the treatment of heart failure, as reviewed elsewhere [5,32,33,34,35]. Overall, although disease progression is complex, dynamic, and patient-specific, immune responses and immune cell-mediated activation of cardiac fibroblasts are usually the first steps in initiating the ECM remodeling process after myocardial infarction. In detail, in response to heart injury, cardiomyocytes undergo apoptosis and release DNA and cellular proteins into the extracellular space that serve as damage-associated molecular patterns [36]. These signals are sensed by innate immune cells, which in turn produce and secrete a variety of pro-fibrotic factors to trigger fibroblast activation. Activated cardiac fibroblasts, referred to as myofibroblasts, are the central cellular effectors in cardiac fibrosis and are characterized by excessive production of ECM components and their smooth muscle cell-like contractile properties obtained by de novo synthesis of alpha-smooth muscle actin-containing stress fibers [37]. Intriguingly, recent studies have shown that different cardiac fibroblast subtypes are present in diseased tissue and undergo temporal variation at the time of injury [38,39,40,41,42]. For example, Ruiz-Villalba et al. identified a unique subset of cardiac fibroblasts that express high levels of collagen triple helix repeat containing 1 after myocardial infarction in mice [41], and Fu et al. have described a subpopulation of cardiac fibroblasts, the matrifibrocytes, that support the mature scar [42]. In addition to cardiac fibroblasts, a growing body of evidence suggests that macrophages are also key mediators of cardiac repair, playing a critical role in orchestrating pro-inflammatory processes immediately after injury (macrophage M1 phenotype) and participating in tissue remodeling by stimulating cardiac fibroblast activation (macrophage M2 phenotype) [43,44]. A fine regulation between the M1 and M2 subtypes is required to achieve a proper resolution of the initial inflammatory response and ensure effective cardiac remodeling. In the context of cardiac fibrosis therapy, EV-based products could act either early after myocardial infarction by stimulating pro-inflammatory M1 macrophages to differentiate into an anti-inflammatory M2 macrophage-like phenotype to attenuate chronic inflammation or at a later time by reducing pro-fibrotic M2 macrophages to alleviate progressive cardiac fibrosis [45,46]. In general, to be reasonable candidates for the treatment of cardiac fibrosis in clinical practice, we propose that EV-based products should meet at least some of the requirements listed in Table 1. Certain aspects have already been demonstrated, for example, for EVs derived from mesenchymal stromal cells [47,48,49,50,51,52]; however, due to our limited understanding of EV properties, it is currently challenging to satisfy all of these aspirations.

Among the variety of molecules encapsulated in EVs that can modulate cardiac fibrosis, regulatory microRNAs (miRs) have been of particular interest in recent years. For example, mesenchymal stromal cell-derived EVs carrying miR-19a, miR-22, miR-29, miR-133, and miR-210 have been shown to reduce cardiac fibrosis during heart regeneration and repair in pre-clinical trials [53,54,55]. Similarly, Ibrahim et al. reported that cardiosphere-derived EVs with enhanced levels of miR-92a attenuated cardiac fibrosis and improved survival in a mouse model of myocardial infarction [56]. However, unlike traditional pharmacological interventions that use single molecules with limited mechanisms of action, EVs deliver not just one miR, but a cocktail of multiple miRs that affect specific cells and tissues in numerous and coordinated ways. Therefore, a deeper understanding of their mechanism of action, potential targets, and possible side effects is desirable prior to the clinical implementation of EV-based products.

## 4. How Can the Therapeutic Efficacy of EV-Based Products Be Measured?

When evaluating the therapeutic efficacy of EV-based products for the treatment of cardiac fibrosis, a major challenge in translating clinical trials into practice is the reliance on so-called surrogate endpoints, such as a significant reduction in infarct size [57]. Other measurable parameters used as surrogate endpoints include left ventricular function, perfusion defects, patient functional status, and quality of life [58,59]. However, while they may not always be reliable indicators of more definitive endpoints, such as mortality, they can at least provide early insight into the therapeutic efficacy of EVs and streamline their development. 

The current gold standard for the diagnosis and evaluation of diffuse cardiac fibrosis is endomyocardial biopsy [60]. However, the invasive nature of the procedure, which is uncomfortable and risky for the patient, has a propensity for sampling error, and is not able to quantify the fibrotic burden of the entire myocardium, limits its use in daily clinical practice. Instead, cardiac fibrosis is more commonly assessed non-invasively and indirectly by measuring cardiac function or by visualizing macroscopic changes in the heart using echocardiography, computed tomography, and cardiac magnetic resonance (CMR) imaging [61]. In particular, late gadolinium enhancement on CMR imaging is a powerful technique for locating and quantifying regions of reparative fibrosis in the heart [62,63,64,65]. However, it is an expensive method that requires considerable skill in image acquisition and analysis and often suffers from poor image quality due to heart rate fluctuations and gadolinium washout during the relatively long acquisition time [66]. More recently, with the advent of novel T1 mapping techniques, reliable assessment of reactive cardiac fibrosis using CMR imaging has become possible [67]. However, the lack of standardization, leading to difficulties in inter-center comparisons, is a major barrier to its widespread adoption [68]. Blood biomarkers, while useful in the early detection of heart failure, remain the only indirect tools for the assessment of cardiac fibrosis [69,70]. For example, elevated serum levels of carboxyl-terminal pro-peptide of type I collagen and amino-terminal pro-peptides of types I and III collagen indicate increased collagen turnover, a marker of fibrotic changes and cardiac repair [71,72]. Unfortunately, none of the routinely used techniques meets all the requirements to determine the degree of cardiac fibrosis and monitor changes after treatment and, therefore, a combination of histological staining, imaging, and biomarker studies is usually needed [73]. Furthermore, it is important to note that the above methods reflect an increase in ECM components rather than the main drivers of cardiac fibrosis: myofibroblasts. Among others, one of their characteristics is the expression of fibroblast activation protein (FAP), a membrane-anchored peptidase [74]. Recently, FAP-targeting radiotracers have been developed that reliably bind and stain FAP, making FAP-specific positron emission tomography-computed tomography a promising non-invasive imaging technique to measure relative FAP density [75,76,77]. 

In summary, there is still a need for safe, reliable, and most importantly, non-invasive tools for routine use to monitor the progression of cardiac fibrosis in general and evaluate changes after administration of EV-based products, in particular in order to assess their therapeutic efficacy.

## 5. How to Deliver High-Quality EV-Based Products?

Despite the increasing attention on EV-based therapeutics and their potential for the treatment of cardiac fibrosis, there are still some limitations that hinder their clinical translation. One of the biggest challenges is the lack of reliable technologies for the large-scale production of EVs under good manufacturing practice (GMP) conditions that allow for high batch-to-batch consistency, purity, and performance. International groups such as the ISEV have established guidelines and protocols for standardized practices, but to date, there is no uniform approach [78,79,80,81,82,83]. 

The first step in the EV manufacturing process is to select the origin of EVs, as they can be derived from either cellular or non-cellular (e.g., body fluids) sources [84,85]. Numerous studies, including clinical trials, have focused on EVs derived from native, unmodified cells, and mesenchymal stromal cell-derived EVs have been at the forefront of these studies [86,87]. Aspects of cell culture that may affect cell status include the type of culture system, the media and supplements used, and the culture condition parameters. Alterations can result in changes in cell state and growth, thus potentially affecting the composition and therapeutic efficacy of the derived EVs. Therefore, culture conditions as well as the metabolic activity and cell number should be monitored regularly, for example by cell viability and proliferation assays [88,89]. 

The second step is EV secretion by the cultured cells into the surrounding cell culture medium, which can be either spontaneous or induced [90]. Spontaneous EV production is chosen to preserve the basal characteristics of the cells. In this case, the entire cell culture medium is replaced by an EV-depleted medium to obtain only the EVs secreted by the target cells under physiological conditions. In contrast, for induced EV production, cell culture under serum starvation conditions is the simplest strategy to increase EV yield, but it may affect cell behavior and, consequently, EV composition and quality. Other methods include pH value change, temperature shift, hypoxia, and additives in the culture medium, as well as chemical induction and physical stimulation, as discussed elsewhere [90]. In any case, all approaches to induce EV secretion from cultured cells must be proven to be safe and GMP-compliant. 

In the third step, EVs are isolated from the cell culture medium; however, there is no consensus on the optimal isolation strategy. In fact, different research laboratories use different protocols to isolate EVs, including, for example, differential ultracentrifugation, size exclusion chromatography, polymer-based precipitation, and immunoaffinity separation [91,92]. Each of these methods has its own advantages and limitations, and there is wide variability in efficiency and purity [93]. From a regulatory perspective, purity concerns are of paramount importance, but EV-based products cannot currently be produced in a completely pure form. The end product of standard isolation methods is only referred to as an EV-enriched preparation that contains other components, such as protein aggregates [94,95]. In addition, regardless of the isolation method, a layer of biomolecules may be adsorbed on the surface of EVs, the so-called corona [96,97]. The corona cloaks the surface of EVs and can cover surface receptors, subsequently affecting their interactions with recipient cells [98]. Although research groups have shown that additional purification of EVs significantly reduces the number of proteins and nucleic acids in EV preparations, corona remodeling may have a major impact on downstream biological effects and thus the efficacy of EV-based products [99]. 

After isolation, in-depth characterization of EV preparations is an important aspect to ensure safe and effective clinical translation. Currently, EVs are identified using multiple complementary methods to determine particle number, size, morphology, surface markers, functionality, and cargo composition. It is generally recommended to perform nanoparticle tracking analysis for particle quantification and size estimation [100]. To evaluate the structure and distinguish EVs from non-EV particles, transmission electron microscopy is currently the most used method [101]. In addition, it is recommended to demonstrate the presence of commonly reported EV markers such as transmembrane proteins (CD9, CD63, CD81), heat shock proteins (Hsp70, Hsp90), or membrane fusion proteins (Annexin, TSG101) using standard antibody-based techniques (e.g., Western blot, enzyme-linked immunosorbent assay, or flow cytometry) [102,103]. In order to demonstrate EV functionality, it is important to test its uptake into recipient cells, for example, by using fluorescently labeled EVs [104,105]. EV cargo profiling is a key strategy for understanding the effects of EVs. Technologies central to this effort include targeted and untargeted mass spectrometry, proteomics, lipidomics, and high-throughput RNA sequencing [106,107,108,109]. Future developments in machine learning may further advance their use. 

Another important consideration in the development of EV-based products is their preservation and storage [23,110]. Although there are no standardized storage protocols available, studies have shown that storage of EVs at −80 °C in single-dose aliquots for up to 7 months does not affect their potency and activity [83,111]. 

In summary, due to the complexity of EVs, commercialization of large-scale manufacturing of EV-based therapeutics requires a technologically superior facility, a robust quality management system, and a GMP-compliant technology in order to deliver high-quality, well-characterized products to patients. 

## 6. What Safety Issues Must Be Considered for EV-Based Products?

In addition to efficacy and quality aspects, safety is of paramount importance for the clinical implementation of EV-based products. In fact, in early clinical trials, when EVs are first used in humans, safety is the priority. Potential risks associated with EV-based therapeutics include (i) undesired distribution in the body, (ii) unwanted immune reactions, such as allergy and rejection, (iii) side effects of components other than EVs administered concomitantly, (iv) involvement in cancer progression and metastasis, especially when applied multiple times over a long period of time, (v) transmission of infectious diseases through microbial contamination of EV preparations, and (vi) variability in efficacy and quality. 

Overall, there are two methods of EVs delivery: intramyocardial injection, which is efficient but invasive, and intravenous injection, which is less invasive but results in low cardiac retention. Previous research has shown that intravenously applied EVs have a limited half-life in the blood and are rapidly cleared in the liver, lungs, and spleen [112,113]. In fact, excessive retention of EVs in the liver not only affects their bioavailability but also increases the risk of developing liver damage. A better understanding of the pharmacokinetics and pharmacodynamics of EV-based products is key to their clinical translation but is hampered by the limitations of our current animal models. First, the animals used are typically young, whereas heart failure patients with cardiac fibrosis are older. Second, methods to detect EVs in tissues and organs require a substantial accumulation of fluorescent or radiolabeled EVs, which is a challenging task and may not correspond to physiological conditions. Third, the biodistribution of EVs varies depending on the isolation method used; for example, intravenously administered EVs isolated by ultracentrifugation plus liquid chromatography show less accumulation in the lungs than EVs isolated by ultracentrifugation alone [114]. Compared to intravenous delivery, intramyocardial administration of EVs may prolong the lifespan of EVs in the heart, but the procedure is more complex and carries a higher risk of complications. 

Numerous studies have shown that EVs are unlikely to induce an immune response, but only a few research groups have thoroughly evaluated their potential for toxic or immunogenic effects. However, this is particularly important because therapeutic EVs, mostly derived from human cell lines, are initially tested in animal models throughout pre-clinical development. In one of the most thorough investigations into the immune response of human EVs in animal models, Zhu et al. showed in a mouse model that human embryonic kidney 293 T cell-derived EVs had no toxic effects, and immune markers were not significantly altered over a 3-week period [115]. 

Another safety issue with EVs is related to the lack of clarity about their cargo. A recent study of known miR targets has revealed that miRs found in mesenchymal stromal cell-derived EVs may also play a critical role in the tumor biology of various cancers [116]. Given that EVs have a half-life of less than 24 h [117], a single administration of EV-based products may not be sufficient to target cardiac fibrosis, and multiple doses would be required. However, this approach could lead to the accumulation of oncogenic miRs in patients with early-stage cancers that were not detected prior to treatment. It would, therefore, be important to screen patients in advance to avoid effects that could favor or even worsen existing tumors. 

In addition to the characterization of EVs, the absence of microbial contamination is an important issue before EV-based products can be utilized in clinical settings. Due to the relatively small volume of EV preparations, filtration sterilization could be performed at the end of the isolation process [118], but standard measures to ensure product sterility are still lacking. Regarding viral safety, given the similarities between EVs and viral particles in terms of size and composition, EV-producing cells should be carefully monitored for a viral infection at the beginning of EV production, and EV preparations should be tested at all relevant manufacturing steps. 

In conclusion, future studies should focus on conducting a thorough and long-term safety evaluation of EVs, which could also help to determine their safe and therapeutic doses for clinical use.

## 7. Conclusions

**Figure 1:**
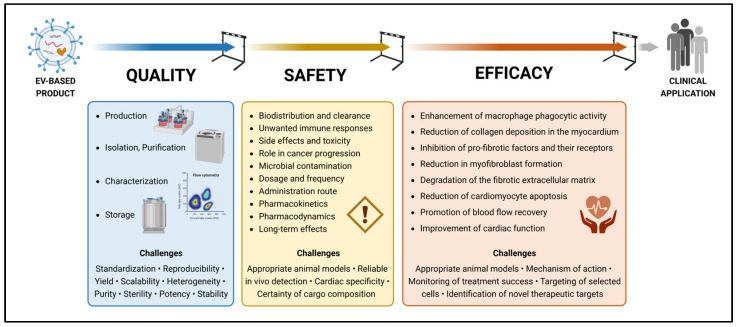
Hurdles to clinical application of EV-based products for the treatment of cardiac fibrosis. Created with BioRender.com.

We believe that EVs represent the next frontier in cell-free therapy; however, this research is still in its infancy, and there is a long way to go before clinical application (Figure 1). One of the most critical challenges for EV-based therapy in heart failure patients is the cardiac specificity and retention of EVs. Most of the currently reported therapeutic effects of EVs on cardiac diseases are based on the direct administration of EVs into the myocardium or pericardial cavity, which is too invasive for routine clinical application. To achieve the therapeutic effects of EVs by intravenous administration, further research is needed to develop efficient EV delivery methods. In addition to that, there are more hurdles to overcome. First, standardized and quality-controlled GMP-compliant methods must be optimized on an industrial scale for the reproducible production of homogeneous EV-based therapeutics. Second, gold standards must be defined to characterize the composition and purity of EV preparations. However, given the difficulties in isolating a uniform EV population, a possible strategy would be to prioritize therapeutic efficacy over purity. Downgrading the regulation of regenerative medicine products could accelerate their clinical implementation, but it is imperative that their safety profile is fully evaluated. Third, appropriate pre-clinical in vivo models must be developed to optimize EV dosing, including the definition of appropriate timing and frequency of application. In particular, in vivo imaging of EVs is required to quantify the number of EVs delivered to damaged cardiac tissue. Fourth, innovative tools must be developed to monitor the progression of cardiac fibrosis after the administration of EV-based products. Fifth, novel therapeutic targets must be identified to selectively reduce or even reverse cardiac fibrosis without any side effects. Timely interdisciplinary studies are needed to address all these challenges. Otherwise, EVs will remain on the laboratory bench, where they show great promise in reducing cardiac fibrosis, and never make it to the bedside.

## Figures and Tables

**Table 1:** Proposed features of EV-based therapy for the treatment of cardiac fibrosis.

(i)	Priming of immune cell phagocytic signaling for efficient clearance of dead cells
(ii)	Limiting cardiac fibrosis by reducing collagen deposition in the myocardium
(iii)	Inhibiting pro-fibrotic factors and their receptors
(iv)	Reduction in myofibroblast formation in the heart
(v)	Direct degradation of the fibrotic ECM in the myocardium
(vi)	Cardioprotection by reducing apoptosis of cardiomyocytes and other cell types
(vii)	Promotion of blood flow recovery by increasing microvascular density
(viii)	Improvement of cardiac function

## Data Availability

No new data were created or analyzed in this study. Data sharing is not applicable to this article.
